# Formaldehyde in Hospitals Induces Oxidative Stress: The Role of *GSTT1* and *GSTM1* Polymorphisms

**DOI:** 10.3390/toxics9080178

**Published:** 2021-07-30

**Authors:** Federica Ghelli, Valeria Bellisario, Giulia Squillacioti, Marco Panizzolo, Alfredo Santovito, Roberto Bono

**Affiliations:** 1 Department of Public Health and Pediatrics, University of Turin, 10126 Turin, Italy; federica.ghelli@unito.it (F.G.); valeria.bellisario@unito.it (V.B.); giulia.squillacioti@unito.it (G.S.); marco.panizzolo@unito.it (M.P.); 2 Department of Life Sciences and Systems Biology, University of Turin, 10123 Turin, Italy; alfredo.santovito@unito.it

**Keywords:** formaldehyde, 15-F_2t_-isoprostane, malondialdehyde, TNFα, *GSTT1*, *GSTM1*, occupational health

## Abstract

Despite the toxicity and health risk characteristics of formaldehyde (FA), it is currently used as a cytological fixative and the definition of safe exposure levels is still a matter of debate. Our aim was to investigate the alterations in both oxidative and inflammatory status in a hospital working population. The 68 workers recruited wore a personal air-FA passive sampler, provided a urine sample to measure 15-F_2t_-Isoprostane (15-F_2t_-IsoP) and malondialdehyde (MDA) and a blood specimen to measure tumour necrosis factor α (TNFα). Subjects were also genotyped for *GSTT1* (Presence/Absence), *GSTM1* (Presence/Absence), *CYP1A1* exon 7 (A > G), and *IL6* (−174, G > C). Workers were ex post split into formalin-employers (57.3 μg/m^3^) and non-employers (13.5 μg/m^3^). In the formalin-employers group we assessed significantly higher levels of 15-F_2t_-IsoP, MDA and TNFα (<0.001) in comparison to the non-employers group. The air-FA levels turned out to be positively correlated with 15-F_2t_-IsoP (*p* = 0.027) and MDA (*p* < 0.001). In the formalin-employers group the MDA level was significantly higher in *GSTT1* Null (*p* = 0.038), *GSTM1* Null (*p* = 0.031), and *CYP1A1* exon 7 mutation carrier (*p* = 0.008) workers, compared to the wild type subjects. This study confirms the role of FA in biomolecular profiles alterations, highlighting how low occupational exposure can also result in measurable biological outcomes.

## 1. Introduction

The exposure to airborne pollutants can result in harmful health effects, depending on the chemical agents, the time of exposure, and the individual susceptibility [1]. Several studies have focused on hospital workers and their occupational risks [1,2,3,4,5], e.g., operating theatre nurses and pathologists, including those who are chronically exposed to formaldehyde (FA) [6,7,8].

FA inhalation has been associated with several toxic effects: low exposure levels (0.1 ppm) can irritate the eyes, nose and upper respiratory airways, while high concentrations may result in pulmonary function impairment and asthma [9,10]. Prolonged exposure to FA has been also associated with nasopharyngeal cancer [11,12] and leukaemia [12,13], and there is limited evidence of a possible association of this compound with sinonasal cancer [14]. In this context, the International Agency for Research on Cancer (IARC) classifies FA as “carcinogenic to humans (Group 1)” [15].

Despite its harmful characteristics, FA is still widely used in pathology wards, where it has been used as fixative for almost 100 years due to its unique ability in preserving cell and tissue morphology [16,17]. Hence, the long-standing concerns over the adverse health effects related to FA exposure [18] in this population.

Workers employed in settings where FA or FA-containing products are made or used may be exposed to such chemicals, mainly by inhalation or skin contact [19]. In order to protect their health, the American Conference of Governmental Industrial Hygienists (ACGIH) updated in 2016 the threshold limit of exposure. Indeed, after having recommended only a threshold limit value-ceiling (TLV-C) of 370 μg/m^3^ since 1992, the limits were updated introducing a “time-weighted average” threshold limit value (TLV-TWA) at 120 μg/m^3^ and a “short-term exposure limit” (TLV-STEL) at 370 μg/m^3^ [20]. The European Union (EU) Scientific Committee for Occupational Exposure Limits (SCOEL) proposed a FA 8-h OEL of 370 μg/m^3^ and a 15-min STEL of 740 μg/m^3^ [21].

The EU Carcinogens and Mutagens Directive of 2019 recently set a Binding Occupational Exposure Limit (BOEL) at 0.37 mg/m^3^ (0.3 ppm) for long-term exposure (8-h time-weighted average) and of 0.74 mg/m^3^ (0.6 ppm) for 15-min exposure. Moreover, a specific 8-h limit value of 0.62 mg/m^3^ (0.5 ppm) was introduced for the healthcare, funeral and embalming sectors, which will be valid until July 2024 [22].

A recent review on the management of FA pollution in pathology wards [23] highlighted that most studies had suggested improving the exhaustion ventilation systems and to use appropriate personal protective equipment (PPE). In the same regards, other research demonstrated the effectiveness of technological approaches, such as photocatalyst and vacuum sealing technologies, alongside with the use of alternative fixatives to formalin [23]. However, due the current lack of an effective alternative chemical, the use of formalin is still needed, making it necessary to arrange various measures to control workers’ exposure, such as the isolation of activities producing greater emissions and the adoption of new standard procedures in order to reduce the number of samples soaked into formalin [22].

The possible mechanisms underlying the FA-induced long-term effects include inflammation, oxidative stress (OS), and apoptosis [24]. Indeed, FA is a powerful trigger of inflammation in the lower airways [25]; several signalling mechanisms, such as the MAPK and NF-κb pathways, have been proposed alongside the FA-induced increase in intracellular reactive oxygen species (ROS) [26]. This condition of imbalance between an excess of oxidant compounds and insufficient antioxidant defences [27], can lead to oxidative damage to biomolecules, including proteins, lipids, and nucleic acids, for example through lipid peroxidation [28]. The alteration of oxidative status is frequently studied through specific biomarkers, such as malondialdehyde (MDA) and F_2_-isoprostanes (F_2_-IsoPs) as measure of lipid peroxidation [29,30,31]. In order to protect themselves against hostile oxidative environments, living organisms evolved several antioxidant defenses, including antioxidant enzymes as well as non-enzymatic ROS scavengers [32]. One of the main non-enzymatic antioxidants is glutathione (GSH), which is able to scavenge ROS and thus decrease OS [26,33]. This compound is also involved in FA-absorbed oxidation, since dehydrogenation of FA requires GSH [34,35].

ROS and antioxidants could also influence the immune system [36]. Indeed, the OS condition could lead to an increase in airway and systemic inflammation associated either to T_H_1 or T_H_2 cytokine production [37]. Specifically, interleukin (IL)-6 and tumour necrosis factor-alpha (TNF-α) play a key role in the immune and inflammatory response and several studies have evaluated the alteration of their levels following FA exposure, albeit with contrasting results [38,39,40,41,42].

Simultaneously, several genetic pathways have evolved for minimizing the effects of environmental exposures [43]. The heritable variability of these genes may be associated with an altered efficiency of the processes in which they are involved [43]. Among these, the enzymes belonging to the glutathione S-transferase (GST) and cytochrome P450 (CYP) families take part in a two-step detoxification process of a wide spectrum of environmental xenobiotics [44,45]. Moreover, the vast majority of GSTs’ functions are associated with detoxification or anti-oxidation processes: structural changes in these molecules can lead to a high variation in their enzymatic activity, resulting thus in OS intensification which can lead to an increase in susceptibility to chronic diseases such as hypertension and cancer [46]. Similarly, cytokine genes can also be polymorphic, resulting in an alteration of the overall expression and secretion of these molecules, partly explaining the inter-individual differences in immune responsiveness [1].

Thus, the aim of this study is to evaluate the systemic oxidative and inflammatory status alteration in a hospital working population routinely exposed to air-FA. Moreover, to better understand the role of inter-individual differences in the exposure-related outcomes, all participants were genotyped for polymorphisms involved in xenobiotic metabolism and in cytokine production. We selected the following polymorphisms: *CYP1A1* exon 7 (A > G), which is involved in phase I metabolic pathways, *GSTT1* (Presence/Absence) and *GSTM1* (Presence/Absence), which are involved in phase II reactions, and, finally, *TNF-α* (−308, G > A) and *IL-6* (−174, G > C) to evaluate the possible role of an alteration in the production of cytokines involved in long-term inflammation processes. Indeed, our interest is to highlight the susceptibility biomarkers relevant in occupational studies and, more specifically, their role in influencing the oxidative and inflammatory status.

## 2. Materials and Methods

### 2.1. Study Population

Sixty-eight workers variously exposed to FA (nurses, health care assistants, laboratory technicians and pathologists) were recruited at “Città della Salute e della Scienza” (Turin, Italy), during the same sampling campaign described in our previous study [7]. Only those subjects who agreed to give a blood sample were considered eligible for the purpose of this study. In accordance with the 2013 Declaration of Helsinki, all subjects received detailed information about the project and gave their informed consent before the participation. The research protocol was approved by the Bioethical Committee of University of Turin.

Each subject: (1) wore a personal air-FA sampler for an 8-h working shift; (2) completed a standardised questionnaire; (3) provided biological samples for the quantification of OS and inflammatory biomarkers: a spot of urine for the quantification of OS biomarkers (15-F_2t_-IsoP, and MDA) and creatinine (CREA), and a fresh blood sample to measure TNFα and single nucleotide polymorphisms (SNPs) frequency.

Workers involved in this study were employed in tasks involving FA use in rotation and were classified ex post as “formalin-employers” or “non-employers”, according to the use of formalin by each volunteer during the sampling day. The exposure level measured in the formalin-employers group could be considered as an approximation of the mean exposure level of the period in which they employed FA-based chemicals. Conversely, the exposure levels measured in the subject group not employing formalin could be representative of the general indoor air-FA level.

### 2.2. Questionnaire, Personal Air-FA Exposure Assessment, and Biological Measurements

The questionnaire and its administration, air-FA sampling and the urine spot collection and storage have been previously described [7].

Briefly, a standardised questionnaire was administered to all workers to collect information about individual factors (sex, age, BMI), working conditions (task, FA exposure, working years) and lifestyle factors (smoking).

To assess the individual (personal) exposure to air-FA, each volunteer wore a radial diffusive personal passive sampler during an 8-h working shift.

During the same day, each subject also provided a specimen of blood and a spot of urine in order to obtain systemic measurement of the studied alterations, given the short half-life of FA, which is rapidly metabolised after entering in the body.

#### 2.2.1. Personal Air-FA Exposure

Given the high variability in FA exposure according to the different tasks of the subjects enrolled, the individual exposure was quantified, for each subject, using a radial symmetry diffusive sampler (Radiello^®^, ICS Maugeri SpA, Pavia, Italy) (https://radiello.com/, accessed on 8 June 2021), as described in previous studies [47,48]. The sampler was clipped near the volunteers’ breathing zone during a whole working shift, in order to assess the FA concentration in the air inhaled by each worker.

Briefly, the photoprotective diffusive body of the sampler, operating in diffusive mode, was equipped with sorbent cartridges consisting in a stainless-steel mesh tube filled with Florisil™ coated with 2,4-dinitrophenylhydrazine (2,4-DNPH). The reaction between airborne aldehydes and 2,4-DNPH leads to the production of the corresponding 2,4-dinitrophenylhydrazone, that can be extracted by 2 mL of acetonitrile (Merck, Milan, Italy), directly inserted in each cartridge tube and then stirred for 30 min. The solution obtained was subsequently filtered and analysed by reverse-phase HPLC with a UV detector set at a wavelength of 365 nm. 10–50 μL of the solution were injected and eluted at flow of 1.9 mL/min. An isocratic elution was performed with acetonitrile/water 38:62 *v*/*v* for 10 min, up to acetonitrile/water 75:25 *v*/*v* in 10 min, and reverse gradient to acetonitrile/water 38:62 *v*/*v* in 5 min. FA was quantified by high performance liquid chromatography (HPLC) according to the NIOSH method No. 2016. The quantification limit was twice the detection limit: 0.10 μg/mL and 0.05 μg/mL, respectively. The CV values were <5%.

#### 2.2.2. OS Measurement

15-F_2t_-IsoP and MDA urinary concentrations were quantified by competitive E.L.I.S.A. kit (Urinary Isoprostane ELISA Kit, Oxford Biomedical Research, Rochester Hills, MI, USA and TBARS assay kit, Abnova, Taipei, Taiwan, respectively) performed according to the manufacturer’s instructions. Urinary creatinine was determined by the kinetic Jaffé procedure [49] to normalise the urinary excretion rate of 15-F_2t_-IsoP.

Briefly, to measure the total fraction 15-F_2t_-IsoP, urine samples were incubated for 2 h at 37 °C with β-glucuronidase and then mixed with an enhanced dilution buffer to eliminate interferences due to non-specific binding. The assay measures the 15-F_2t_-IsoP in samples or standards competing with 15-F_2t_-IsoP conjugated to horseradish peroxidase (HRP) for binding to a polyclonal antibody specific for 15-F_2t_-IsoP coated on the microplate through colour development when the substrate is added. The colour intensity is inversely proportional to the amount of unconjugated analyte in samples or standards. The LOD was 0.08 ng/mL.

The Thiobarbituric Acid Reactive Substances (TBARS) assay, instead, is based on the quantification of the MDA-TBA adduct formed by MDA and TBA under high temperature (99–100 °C) and acidic conditions. The reaction between one molecule of MDA and 2 molecules of 2-thiobarbituric acid leads to the formation of a chromophore and can be measured colorimetrically at 530–540 nm.

#### 2.2.3. Blood Sample Collection and Inflammatory Biomarkers

Blood samples were obtained by venepuncture (5–10 mL) and collected in heparinised Vacutainer^®^ (Becton, Dickinson and Company, Franklin Lakes, NJ, USA).

TNF-α was quantified using a Human Soluble Cytokine Receptor Panel kit (MILLIPLEX^®^ MAP, Merck, Milan, Italy). This assay is based on the Luminex xMAP^®^ technology, a multiplex technology capable of performing immunoassays on the surface of fluorescent-coded magnetic beads known as MagPlex^®^-C microspheres. Luminex uses proprietary techniques to internally colour-code microspheres with two fluorescent dyes. Through precise concentrations of these dyes, distinctly coloured bead sets of 500 5.6 μm polystyrene microspheres or 80 6.45 μm magnetic microspheres coated with a specific capture antibody are created. After the bead captures an analyte from a test sample, a biotinylated detection antibody is introduced. Streptavidin-PE conjugate, the reporter molecule, is added to the mixture to allow the reaction conclusion on the surface of each microsphere. Each microsphere is identified. Our results were quantified based on fluorescent reporter signals [50].

#### 2.2.4. DNA Extraction and Genotyping

Genomic DNA was extracted from white blood cells isolated with the Ficoll separation method and stored at −140 °C. Vials containing whole blood were centrifuged at 1000 rpm for 10 min, with the centrifuge brake set to off. Plasma was aspired with a venturi pump, leaving about 1–2 mL of plasma in the vial. The remaining content was transferred in a Falcon tube and the pellet was diluted 1:2 with RPMI-1640 medium (Thermo Fisher Scientific, Inc., Waltham, MA, USA) or PBS (Thermo Fisher Scientific, Inc., Waltham, MA, USA) previously heated in a thermostatic bath. The tube content was then transferred drop by drop, at least in the first steps, in a clean Falcon tube containing 10–15 mL of Biocoll solution previously heated in a thermostatic bath. Falcon tubes were centrifuged for 25–30 min at 1700–1800 rpm with the centrifuge brake off. The opalescent ring formed containing lymphocytes and monocytes was transferred in a clean tube. The washing step was performed with RPMI-1640 medium, adding 5 mL of medium drop by drop, mixing by inversion 1–2 times and then adding medium up to 30–50 mL. Tubes were centrifuged for 8–10 min at 1400 rpm with centrifuge brake off. The supernatant was removed by aspiration with a Venturi pump and the pellet was gently re-suspended by hitting the bottom of the tube. If the supernatant was turbid, RPMI-1640 medium was again added in tubes with samples up to 15–20 mL; samples were mixed by inversion and centrifuged 8–10 min at 1200 rpm with the centrifuge brake off. The supernatant was removed, the pellet re-suspended and 1 mL of freezing medium (50% inactivated FBS (Thermo Fisher Scientific, Inc., Waltham, MA, USA), 40% RPMI-1640 medium, 10% DMSO (Merck, Milan, Italy)) was then added drop by drop to each sample. Cells were resuspended, and 1 mL of the sample was transferred in a cryovial. Vials were stored at −80 °C and then at −140 °C. DNA extraction was conducted by using a salting-out procedure: white blood cells were centrifuged at 14,000 rpm and the pellet was resuspended in a solution consisting of 340 µL white cell lysis buffer (10 mM Tris pH 7.6 (Merck, Milan, Italy); 10 mM EDTA (Merck, Milan, Italy) and 50 mM NaCl (Merck, Milan, Italy)), 10 µL of SDS 10% (Merck, Milan, Italy) and 30 µL of proteinase K (Merck, Milan, Italy). After incubation at 55 °C for 30 min, 200 µL of saturated sodium acetate was added to the solution. The samples were vigorously shaken and centrifuged at 14,000 rpm for 5 min. Subsequently, 0.5 mL of isopropanol for DNA precipitation were added to the supernatant solution and, after centrifugation at 14,000 rpm for 1 min, 0.5 mL of 70% ethanol was added to remove salt from the pellet. After 30–60 min at room temperature, the pellet was resuspended in 50 µL of ultrapure distilled water.

All subjects were genotyped for *GSTT1, GSTM1, CYP1A1* exon 7, TNF-α and IL-6 by using primers and methodologies as described in Table 1. PCR reactions were performed in a 25 μL volume containing about 10 ng DNA (template), with a final concentration of 1X Reaction Buffer (Thermo Fisher Scientific, Inc., Waltham, MA, USA), 1.5 mM of MgCl_2_ (Thermo Fisher Scientific, Inc., Waltham, MA, USA), 5% of DMSO (Thermo Fisher Scientific, Inc., Waltham, MA, USA), 250 µM of dNTPs (Thermo Fisher Scientific, Inc., Waltham, MA, USA), 0.5 μM of each primer, and 1 U/sample of Taq DNA polymerase (Thermo Fisher Scientific, Inc., Waltham, MA, USA). Cycles were set as follows: 35 cycles, 1 min at 95 °C, 1 min at 60–65 °C (depending on the gene polymorphism, Table 1), 1 min at 72 °C, and a final extension step 10 min at 72 °C. Amplification products were detected by ethidium bromide (Merck, Milan, Italy) staining after 3% agarose gel (Società Italiana Chimici, Rome, Italy) electrophoresis.

### 2.3. Statistical Analysis

Statistical analyses were performed by SPSS Statistics 27 (IBM SPSS Statistics, New York, NY, USA) and RStudio (RStudio Desktop 1.2.5042, RStudio Inc., Boston, MA, USA). A Shapiro–Wilk test was performed to assess the normality of the distributions.

Descriptive analyses were performed by using χ^2^ or Fisher tests for categorical variables (sex, smoking, task, working years, and gene polymorphisms) and a Wilcoxon test or t-test for continuous variables (age, BMI, FA, 15-F_2t_-IsoP, MDA, and TNF-α), as appropriate. Non-parametric correlations were assessed by Spearman’s test to investigate the correlation between biomarkers and FA exposure, and between biomarkers themselves. Non-parametric comparison of biological and environmental variables between formalin-employers and non-employers groups were performed by the Mann–Whitney or Kruskall–Wallis tests. Boxplots were drawn to show the difference of MDA levels between formalin-employers and non-employers according to *GSTT1*, *GSTM1*, and *CYP1A1* polymorphisms.

## 3. Results

The general description of the sample was described in the previous paper [7], while the following descriptive analyses are referred to FA exposure (Table 2 and Table 3).

As expected, the difference in FA levels between formalin-employers and non-employers proved to be highly significant (*p* < 0.001). Concerning biomarkers, the comparison between formalin-employers and non-employers revealed a significant alteration in both oxidative and inflammatory status. Specifically, significant differences were found in 15-F_2t_-IsoP (*p* < 0.001), MDA (*p* < 0.001), and TNF-α (*p* = 0.023). Conversely, no differences were found concerning confounding factors such as gender, age, BMI, tasks, working years and smoking habits, nor in the frequency of polymorphisms’ distribution.

The air-FA concentration was significantly and positively correlated with 15-F_2t_-Isop (Spearman’s rho = 0.269, *p* = 0.027) and MDA (Spearman’s rho = 0.647, *p* < 0.001). Among biomarkers a correlation was found between 15-F_2t_-Isop and MDA (Spearman’s rho = 0.471, *p* < 0.001) and MDA and TNF-α (Spearman’s rho = 0.299, *p* = 0.013).

**Figure 1:**
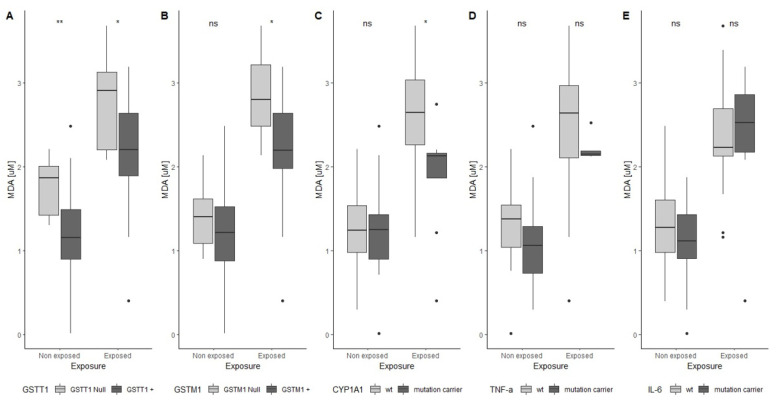
Boxplot of MDA levels in formalin-employers and non-employers according to the five polymorphisms analysed: (**A**) *GSTT1*, (**B**) *GSTM1*, (**C**) *CYP1A1*, (**D**) TNF-α, (**E**) IL-6. In each panel the non-employers data are depicted on the left side, while the formalin-employers data are on the right. In each group, the boxplot in light grey shows the mutation carriers for the selected genes, while the boxplot in dark grey is related to the wt. *: *p* < 0.05, **: *p* < 0.01, ns: not significant, dots represent the outliers.

Regarding the gene polymorphisms, *GSTT1* Null subjects had higher MDA concentration than *GSTT1+* in both formalin-employers and non-employers t = 2.220, *p* = 0.038 and t = 2.827, *p* = 0.007, respectively Figure 1A).

In formalin-employers, higher MDA levels were found in subjects *GSTM1* Null (t = 0.679, *p* = 0.031, Figure 1B) compared to *GSTM1+* and in those who carried at least one mutated allele for the *CYP1A1* gene (U = 20.50, *p* = 0.008, Figure 1C) in comparison with the wt. The additional comparison between the wt and carriers of mutation for the other biomarkers did not provide any significant results. 

## 4. Discussion

FA has been the most popular fixative in histology: the ease in use, the low cost and the variety of techniques that can be performed following fixation are only some of the properties which made widespread the usage of this chemical [56]. Nevertheless, the health and safety risks associated with formalin exposure are still a matter of concern [57].

The main aim of this study was to investigate wide range of outcomes in terms of alteration of the oxidative and inflammatory status related to occupational exposure to FA, considering the possible role of genetic polymorphisms in modulating the individual response.

The FA metabolism includes several oxidation steps that could lead to ROS generation [58], and, in turn, to the alteration of exposed subjects’ biomolecular profiles, as confirmed in this study.

Oxidative stress and inflammatory biomarkers levels must be interpreted as the outcome of the metabolic pathways of the days before sampling.

Among formalin-employers we found a significant alteration in the systemic oxidative status, highlighted by significantly higher urinary concentration of 15-F_2t_-IsoP and MDA than in non-employers. In line with our results, many studies in literature reported interesting data demonstrating systemic adverse effects following FA exposure in occupational settings [4,59,60].

A huge number of studies to date have demonstrated the role of ROS in the pathogenesis of chronic diseases including cancer. ROS sources could be both endogenous, from mitochondria, peroxisomes or inflammatory cell activation, and exogenous, caused by environmental agents, radiations, pharmaceuticals, or industrial chemicals. When an excess of ROS is produced for a long time, resulting in a condition known as OS, cellular structure and functions may be affected by significant damages, which, in turn, may induce somatic mutations and neoplastic transformation [61,62].

The air-FA concentration measured in the workers’ breathing zone resulted positively correlated with levels of 15-F_2t_-IsoP and MDA. These results agree with reports of our previous studies, coming from professional exposures monitoring and toxicology research. Bellisario et al. (2016), particularly, demonstrated a direct role of air-FA exposure on the alteration of the oxidative status, with higher levels of urinary 15-F_2t_-IsoP and MDA in nurses employing FA, although MDA levels were found to be significantly higher only in nurses exposed to liquid FA, in particular, higher FA concentrations [7].

The high MDA levels in formalin-employers are noteworthy, since the interaction between this molecule and nucleic acid bases can lead to several adducts, including the 3-(2-deoxy-β-D-erythro-pentafuranosyl)-pyrimido-[1,2-α]-purin-10(3H)-onedG (M_1_dG) [63]. Specifically, in a study on pathologists’ exposure to FA, the M_1_dG concentration was found to be significantly higher in workers than in the control group, with a dose-dependent relationship associated with exposures exceeding 66 μg/m^3^ [63]. In our study, the median FA concentration measured in the formalin-employers group was 57.3 μg/m^3^. Therefore, we can suppose that our sample, or at least a some of the volunteers enrolled, might be at risk of M_1_dG adducts formation.

Concerning the correlations between OS biomarkers, we also found a significant, positive correlation between MDA and 15-F_2t_-IsoP, about which contrasting reports have been previously published [64,65].

The specific role of FA in triggering airway inflammation remains still unclear [26]. In a study on workers employed in the production and the utilisation of FA-melamine resins, Seow et al. (2015) highlighted a potential association between FA and immunosuppression, suggesting that high exposures may result in subtle immunological alterations [24]. We found a significant difference in TNF-α level, with the higher concentration in the formalin-employers group. This finding is remarkable, since TNF-α is able to trigger the transcription of the nuclear factor kappa B, involved, in turn, in both inflammatory and OS processes [61]. Moreover, these results are in line with the results provided by Oztan et al. (2020), reporting an increase in TNF-α and IL-6 serum levels in workers employed in a fibre manufacturing company exposed to FA [60]. The significant positive correlation we found between TNF-α and MDA levels, might be considered as a possible link between the alteration in both inflammatory and oxidative status, even though contrasting reports can be found [66,67,68]. Our results, thus, could be further evidence of the role of FA in the immune profile alteration.

In this context, the investigation of genetic susceptibilities, and more specifically, the analysis of genetic polymorphisms that may be involved in modulating the individual susceptibility to occupational disease is of utmost importance [43]. Indeed, the results from previous studies and the present study could enable identification of worker subgroups susceptible to specific workplace exposures, and to provide useful inputs to update the current exposure limits [43].

In a recent review, Nielsen et al. (2017) reported that polymorphisms seem not to have any influence on modulating the genotoxic effect due to FA exposure [14]. Despite these premises, in our study we found lower MDA levels in *GSTT1* Null subjects, both in the formalin-employers and non-employers, in formalin-employers *GSTM1* Null and in those carrying at least one mutate allele for the *CYP1A1* exon 7 (A > G) gene.

GSTs are part of a family of phase II metabolising enzymes with a wide variety of biological roles, including cell protection against OS and toxic molecules. These enzymes are able to conjugate GSH to hydrophobic and electrophilic molecules, including carcinogens, drugs, and products of oxidative metabolism, making them less toxic and suitable to further modification and/or elimination [69]. The FA oxidation process requires GSH. Indeed, FA binds quickly and reversibly with GSH right after entering in the body, forming S-hydroxymethylglutathione, which is subsequently oxidised by cytosolic GSH-dependent FA-dehydrogenase. In an alternative pathway, free FA is first oxidised to formate, and then further to carbon dioxide [58]. In vitro studies confirmed that GSH depletion is one of the consequences of FA exposures: FA-induced cytotoxicity was proved to be influenced by intracellular GSH level [70], and, in neurons, FA exposure was found to induce GSH release, with an increase of the GSx amount in the extracellular medium [71]. The null genotype is characterised by a deletion of *GSTM1* and *GSTT1* genes, leading to the absence of the enzymes. The homozygous deletions are, thus, involved in a reduced elimination of carcinogens, and the carrier subjects are more susceptible to oxidative injuries [72]. Some studies have reported the association between deletion of the *GSTT1* and *GSTM1* genes and an increase in lipid peroxidation biomarkers, such as MDA [66,72]. Contrasting results are, instead, reported concerning the association between these two gene polymorphisms and genomic damage in FA exposed workers: some studies found no association [3,73] while Santovito et al. (2017b), in a pathologist sample, found significantly higher frequency of sister chromatid exchanges (SCEs) and chromosomal aberrations (CAs) in *GSTT1* Null subjects. Thus, we can suppose that the lower reduction of MDA levels in *GSTT1+* formalin-employers than in non-employers could be ascribed to FA’s pro-oxidant role, overcoming the enzyme antioxidant activity. The lower MDA concentration found in subjects with at least a mutated allele for the *CYP1A1* was in contrast with results reported in the literature, where the lower MDA levels were found in wt subjects [67,68]. The main limitation of this study is the cross-sectional design, which prevents the possibility of causal inferences. Longitudinal studies should be performed, in order to clarify causes of oxidative and inflammatory status alteration.

The strengths, on the other hand, consist in the quantification of personal exposure of workers enrolled in the study and in the measurement of many different biomarkers representing the different pathways that could be altered following the FA exposure. In addition, having also considered the genetic differences that could have an impact in the responsiveness to harmful exposure allowed us to highlight the importance of considering individual susceptibility in occupational settings.

## 5. Conclusions

This study confirms once more the role of FA exposure in the alteration of both oxidative and inflammatory profiles, highlighting how daily occupational exposure, even at low levels and in compliance with current legislation, can result in measurable biological outcomes. Moreover, this research highlights once more the importance of considering individual susceptibility biomarkers in the characterisation of biological outcomes following occupational exposures. These results, therefore, stress the relevance of preventive measures intended to reduce the FA exposure in workers who must use FA-based compounds during their work, e.g., vacuum systems for pathologists and nurses employed in operating theatres. In this scenario, in terms of public health and preventive strategies, gathering information on the different factors able to influence the biological outcome onset is of utmost importance in order to allow the definition of exposure limits for the safety of workers.

## Figures and Tables

**Table 1:** Description of primer sequence, annealing temperature, methodology employed and PCR product size (bp).

Gene	Polymorphism NCBI	Main Function Protein	Sequence	T (°C)	Methodology	PCR Product Size (bp)	Reference
*CYP1A1* (A > G)	rs1048943	Phase-I metabolic enzyme	5′-AAGACCTCCCAGCGGGCAAT-3′ 5′-AAGACCTCCCAGCGGGCAAC-3′ 5′-CTCTGGTTACAGGAAGCTAT-3′	60	ARMS-PCR	162	[51]
*GSTT1* (Presence/ Absence)	rs1601993659	Phase-II metabolic enzyme	5′-TTCCTTACTGGTCCTCACATCTC-3′ 5′-TCACCGGATCATGGCCAGCA-3′	63	PCR	480	[52]
*GSTM1* (Presence/ Absence)	rs1183423000	Phase-II metabolic enzyme	5-CTGGATTGTAGCAGATCATGC-3′ 5′-CTGCCCTACTTGATTGATGGG-3′	65	PCR	273	[53]
*TNF-α* (−308, G > A) Antisense primer G-sense primer A-sense primer	rs1800629	Pro-inflammatory	5′-TCTCGGTTTCTTCTCCATCG-3′ 5′-ATAGGTTTTGAGGGGCATGG-3′ 5-AATAGGTTTTGAGGGGCATGA-3′	60	ARMS-PCR	184	[54]
*IL-6* (−174, G > C) Antisense primer G-sense primer C-sense primer	rs1800796	Pro-inflammatory	5′-TCGTGCATGACTTCAGCTTTA-3′ 5′-AATGTGACGTCCTTTAGCATG-3′ 5′-AATGTGACGTCCTTTAGCATC-3′	60	ARMS-PCR	190	[55]

**Table 2:** Characteristics of the sampled population presented as formalin-employers and non-employers groups. Differences between formalin-employers and non-employers groups were tested by χ^2^/Fisher tests as appropriate and Wilcoxon tests for categorical and continuous variables, respectively.

Characteristics	Formalin-Employers (*n* = 23)		Non-Employers (*n* = 45)		*p*-Value
	*n* (%)		*n* (%)		
Sex					
Male	5 (21.7)		7 (15.6)		0.522
Female	18 (78.3)		38 (84.4)		
Smoking					
Yes	7 (30.4)		12 (26.7)		0.780
No	16 (69.6)		33 (73.3)		
Task					
Healthcare assistant	4 (17.4)		11 (24.4)		0.133
Nurse	7 (30.4)		20 (44.4)		
Laboratory technician	7 (30.4)		12 (26.7)		
Pathologist	5 (21.7)		2 (4.4)		
Working years					
<5	3 (13.0)		5 (11.1)		0.973
5–10	5 (21.7)		10 (22.2)		
>10	15 (65.2)		30 (66.7)		
	Median	IQR	Median	IQR	
Age (years)	44.00	14.00	49.00	13.00	0.369
BMI (kg/m^2^)	23.44	4.20	23.40	7.32	0.429

**Table 3:** FA exposure, biomarker measurements, and polymorphism frequencies of the sampled population presented as formalin-employers and non-employers groups. Differences between formalin-employers and non-employers groups were tested by χ^2^/Fisher tests as appropriate and Wilcoxon tests for categorical and continuous variables, respectively.

Parameters	Formalin-Employers (*n* = 23)		Non-Employers (*n* = 45)		*p*-Value
	Median	IQR	Median	IQR	
FA (µg/m^3^)	57.3	125.9	13.5	12.7	<0.001
15-F_2t_-IsoP (ng/mg CREA)	16.06	14.98	2.16	2.67	<0.001
MDA (μM)	2.29	0.67	1.25	0.64	<0.001
*TNF-α* (pg/mL)	15.35	16.51	11.59	7.31	0.023
	*n* (%)		*n* (%)		
*GSTT1*					
*GSTT+*	15 (65.2)		38 (84.4)		0.120
*GSTT Null*	8 (34.8)		7 (15.6)		
*GSTM1*					
*GSTM+*	16 (69.6)		34 (75.6)		0.772
*GSTM Null*	7 (30.4)		11 (24.4)		
*CYP1A1*					
wt	15 (65.2)		32 (71.1)		0.782
mutation carrier	8 (34.8)		13 (28.9)		
*TNF-α*					
wt	18 (78.3)		30 (66.7)		0.405
mutation carrier	5 (21.7)		15 (33.3)		
*IL-6*					
wt	15 (65.2)		32 (71.1)		0.782
mutation carrier	8 (34.8)		13 (28.9)		

wt, wild type.

## Data Availability

The data presented in this study are available on request from the corresponding author. The data are not publicly available due to privacy restrictions.
